# Impact of different endometrial preparation protocols on pregnancy outcomes in frozen-thawed embryo transfer cycles among patients ≤35 years with a history of intrauterine adhesion separation surgery: a retrospective cohort study

**DOI:** 10.3389/fendo.2026.1736841

**Published:** 2026-04-28

**Authors:** Huaqing Sun, Mingze Du, Shujuan Guo, Hongwu Qiao, Zhancai Wei, Yichun Guan

**Affiliations:** The Third Affiliated Hospital of Zhengzhou University, Zhengzhou, Henan, China

**Keywords:** endometrial preparation protocol, frozen-thawed embryo transfer, intrauterine adhesion (IUA), post-intrauterine adhesion surgery, pregnancy outcome

## Abstract

**Objective:**

To investigate the impact of three endometrial preparation protocols-hormone replacement therapy (HRT), natural cycle (NC), and down-regulation HRT-on pregnancy outcomes in frozen embryo transfer (FET) cycles among patients with a history of intrauterine adhesion (IUA) separation surgery.

**Method:**

A retrospective cohort study was conducted to analyze the data from FET cycles at reproductive medicine center from January 2017 to December 2023. The patients with a history of intrauterine adhesion separation surgery were classified into three groups: the HRT group (n=285), the NC group (n=200), and the down-regulation HRT group (n=104). Baseline characteristics and reproductive outcomes were compared between the groups. Chi-square tests were used for univariate analysis, and multivariate logistic regression analysis was conducted to adjust for confounding factors.

**Results:**

There were statistically significant differences among the three groups in terms of general charateristics (all P<0.05). The clinical pregnancy rates for the HRT group, NC group, and down-regulation-HRT group were 30.88% (88/285), 47.50% (95/200), and 40.38% (42/104) respectively. The biochemical pregnancy rates were 35.09% (100/285), 50.50% (101/200), and 45.19% (57/104) respectively. The early miscarriage rates were 21.59% (19/88), 13.68% (13/95), and 21.43% (9/42) respectively, and the live birth rates were 22.81% (65/285), 36.50% (73/200), and 30.77% (32/104) respectively. There were statistically significant differences in clinical pregnancy rate, biochemical pregnancy rate and live birth rate among the groups. Following adjusting for confounding factors, the NC group exhibited significantly higher clinical pregnancy rate (aOR=1.627, 95% CI: 1.079-2.453, P = 0.02) and biochemical pregnancy rate (aOR=1.532, 95% CI: 1.020~2.301, P = 0.04) relative to the HRT group. There were no statistically significant differences in live birth rate, and early miscarriage rate (all P>0.05). Similarly, there were no statistically significant differences in clinical pregnancy rate, biochemical pregnancy rate, live birth rate, and early miscarriage rate between the down-regulation-HRT group and the HRT group (all P>0.05).

**Conclusion:**

For patients undergoing FET cycle with a history of IUA separation surgery, the live birth rate is similar between three groups. However, the natural cycle endometrial preparation protocol yielded higher clinical and biochemical pregnancy rates than the HRT protocol, suggesting its potential clinical advantage for these patients.

## 1 Introduction

Intrauterine adhesion (IUA) refers to the partial or complete adhesion of the uterine cavity resulting from damage to the basal layer of the endometrium due to intrauterine procedures or infections. IUA is one of the significant causes of female infertility, recurrent implantation failure, and miscarriage. The primary treatment for IUA is surgical intervention; however, the postoperative pregnancy rate remains suboptimal. In the context of assisted reproduction cycles involving frozen-thawed embryo transfer (FET), different endometrial preparation protocols may affect the synchrony and receptivity of the endometrium, ultimately influencing embryo implantation and development. Currently, common FET endometrial preparation protocols include Hormone Replacement Therapy (HRT), GnRH agonist down-regulation followed by hormone replacement therapy (down-regulation-HRT), and the Natural Cycle (NC). However, there is limited research on whether adjusting the endometrial preparation protocol can improve pregnancy outcomes in this specific population with a history of IUA separation surgery. This study aims to compare the effects of three different endometrial preparation protocols on the pregnancy outcomes of FET in patients with a history of IUA separation surgery, with the goal of providing a basis for individualized clinical treatment.

## 2 Subjects and methods

### 2.1 Research subjects and grouping

This study is a retrospective cohort study analyzing the clinical data of patients who underwent frozen embryo transfer (FET) at our center from January 2017 to December 2023. The inclusion criteria were: ① female age ≤ 35 years; ② history of intrauterine adhesion (IUA) separation surgery; ③ normal uterine cavity morphology as assessed by hysteroscopy; ④cleavage-stage embryo transfer cycles or blastocyst-stage embryo transfer cycles; ⑤cycles with either single embryo transfer or double embryo transfer. Exclusion criteria included: ① cycles where either partner underwent preimplantation genetic testing; ② endometritis, submucosal fibroids, severe uterine malformations, etc.; ③ cycles using donor eggs; ④ patients with endocrine abnormalities, such as polycystic ovary syndrome, Hashimoto’s thyroiditis, hyperprolactinemia, etc.; ⑤ cycles utilizing testicular sperm. Participants were divided into three groups based on the endometrial protocol: the hormone replacement therapy (HRT) group, the natural cycle (NC) group, and the down-regulation HRT group. This study was reviewed by the Ethics Committee of the Third Affiliated Hospital of Zhengzhou University (ethics approval number) and was exempt from obtaining patient informed consent.

### 2.2 Endometrial preparation protocols

HRT Protocol: On the third day of menstruation, perform a transvaginal ultrasound to monitor the thinning of the endometrium after shedding (≤6mm). Based on the previous endometrial thickness during the ovulation phase, administer Progynova at a dosage of 2mg twice or three times daily orally. During the hormone replacement cycle, in addition to estrogen medication, add enteric-coated aspirin at 50mg once daily orally. When the endometrial thickness reaches ≥7mm and the estrogen exposure time is ≥12 days, the principle is to have E≥100 ng/L (367 pmol/L), but the primary focus is on the endometrial thickness, which can be used to determine the conversion of the endometrium. Starting from the endometrial transformation day, progesterone support medication was administered: progesterone gel 90 mg once daily intravaginally, combined with dydrogesterone 10 mg three times daily orally, continued until 14 days post-embryo transfer.

NC protocol: During the month of transplantation, monitor ovulation. When the dominant follicle reaches an average diameter of ≥15mm and the endometrial thickness is ≥7mm,daily transvaginal ultrasound was performed to monitor follicular development. If the follicle develops to more than 18mm, and daily serum levels of LH, E_2_, and P were measured. Provide 10,000 IU of HCG via intramuscular injection when either the mean diameter of the dominant follicle reaches ≥20 mm or the serum LH level exceeds 30 IU/L. The luteal phase support protocol was the same as that used in the HRT regimen.

Down regulation HRT Protocol: In the early follicular phase (days 2–4 of menstruation), administer a long-acting GnRH agonist (Triptorelin) 3.75mg via intramuscular injection. After 28–30 days, test blood levels of LH, FSH, E2, and P, and use transvaginal ultrasound to monitor follicle size to determine if the downregulation criteria have been met (same as the criteria for the superovulation protocol). If the criteria are met, administer hormone replacement therapy with Progynova or Femoston in an artificial cycle, following the same application method as previously described. If the criteria are not met, or if the patient has severe adenomyosis, another dose of the long-acting GnRH agonist (Triptorelin) 3.75mg intramuscular injection may be administered. The luteal phase support protocol was the same as that used in the HRT regimen.

### 2.3 Observation indicators

The primary outcome measure of this study is the clinical pregnancy rate. Secondary outcome measures include the biochemical pregnancy rate, live birth rate, and early miscarriage rate. Clinical pregnancy is determined by the presence of a gestational sac observed via ultrasound 28 days after embryo transfer. The clinical pregnancy rate is calculated as the number of clinical pregnancy cycles divided by the number of embryo transfer cycles, multiplied by 100%. The biochemical pregnancy rate is calculated as the number of biochemical pregnancy cycles divided by the number of embryo transfer cycles, multiplied by 100%. The early miscarriage rate is calculated as the number of early miscarriage cycles divided by the number of clinical pregnancy cycles, multiplied by 100%. The live birth rate is calculated as the number of live birth cycles divided by the number of embryo transfer cycles, multiplied by 100%.

### 2.4 Statistical methods

Data analysis was conducted using R. For measurement data that follow a normal distribution, the results are expressed as mean ± standard deviation (x ± s), and comparisons between groups were made using analysis of variance (ANOVA). For non-normally distributed data, the results are expressed as the median (interquartile range) [M(Q1, Q3)], and group comparisons were performed using the Kruskal-Wallis H test. Categorical data are expressed as rates (%), and comparisons between groups were made using the chi-square test. *Post hoc* power calculations were performed with G*Power. A multivariate logistic regression model was employed to analyze confounding factors related to pregnancy outcomes in patients after IUA correction surgery, calculating the odds ratio (OR) and its 95% confidence interval (CI). A P-value of less than 0.05 was considered statistically significant.

## 3 Result

### 3.1 Baseline characteristics of patients

Based on the inclusion and exclusion criteria, a total of 589 cycles were included, with 285 cycles in the HRT group, 200 cycles in the NC group, and 104 cycles in the down-regulation HRT group. **Table 1** shows the baseline characteristics of patients in the three groups. The BMI and AMH levels in the HRT group were higher than those in the NC group, while the endometrial thickness on the day of transplantation was lower in the HRT group than in the NC group, all with statistically significant differences (all P<0.05). There were no statistically significant differences between the two groups for the other indicators (all P>0.05). The endometrial thickness on the day of transplantation in the HRT group was lower than that in the down-regulation HRT group, with a statistically significant difference (P = 0.002), while there were no statistically significant differences between the two groups for the other indicators (all P>0.05). The AMH and AFC levels in the NC group were lower than those in the down-regulation HRT group, while the endometrial thickness on the day of transplantation and the proportion of single embryo transfers were higher than those in the down-regulation HRT group, all with statistically significant differences (all P<0.05). There were no statistically significant differences between the two groups for the other indicators (all P>0.05).

**Table 1:** Baseline characteristics of patients among three groups.

Item	HRT	NC	Down-regulation HRT	χ^2^/H value	P value
No. of cycles	285	200	104		
Cycle classification(%)				0.13	0.938
IVF	85.6(244/285)	84.5(169/200)	85.6(89/104)		
ICSI	14.4(41/285)	15.5(31/200)	14.4(15/104)		
Female age [year, M(Q1,Q3)]	31.00(29.00-33.00)	32.00(29.75-33.00)	31.00(29.00-33.00)	3.03	0.22
Female BMI [kg/m^2^,M(Q1,Q3)]	23.50(21.50-25.60)	22.77(20.70-24.96)^a^	22.97(21.63-25.10)	7.31	0.026
Duration of infertility[year, M(Q1,Q3)]	3.00(1.00-4.00)	3.00(1.50-4.00)	2.75(1.00-4.00)	0.7	0.704
AMH[pmol/L,M(Q1,Q3)]	18.86(8.75-30.02)	13.75(6.52-22.33) ^a^	20.14(11.34-30.07) ^b^	17.53	<0.001
AFC[M(Q1,Q3)]	16.00(11.00-21.00)	16.00(12.00-19.00)	18.00(14.00-22.25) ^b^	7.66	0.022
Endometrial thickness on transfer day[mm,M(Q1,Q3)]	7.40(6.60-8.20)	8.30(7.30-9.20) ^a^	8.00(7.00-9.00) ^a^	49.08	<0.001
No. of embryo transferred(%)				9.41	0.009
1	62.1(177/285)	68.00(136/200)	50.00(52/104)^b^		
2	37.9(108/285)	32.00(64/200)	50.00(52/104)		
Type of embryos transferred(%)				2.13	0.343
Cleavage stage embryo	30.5(87/285)	24.5(49/200)	28.8(30/104)		
Blastocyst	69.5(198/285)	75.5(151/200)	71.2(74/104)		

χ²: Chi−square test for categorical variables. H: Kruskal−Wallis test for continuous variables. ^<s>a</s>^ represents P<0.05, the other two groups compared with HRT group. ^b^ represents P<0.05, the other two groups compared with NC group.

### 3.2 Pregnancy outcomes with different endometrial preparation protocols

There are statistically significant differences in clinical pregnancy rate, biochemical pregnancy rate, and live birth rate among the three groups (P<0.05). Pairwise comparisons between groups show that the clinical pregnancy rate (30.88% vs. 47.50%, P<0.001), biochemical pregnancy rate (35.09% vs. 50.50%, P = 0.003) and live birth rate (22.81% vs. 36.50%, P = 0.004) in the HRT group are significantly lower than those in the NC group. There are no statistically significant differences between the HRT group and the down-regulation HRT group in clinical pregnancy rates, biochemical pregnancy rates, early miscarriage rates, and live birth rates (P>0.05). Similarly, there are no statistically significant differences between the NC group and the down-regulation HRT group in clinical pregnancy rates, biochemical pregnancy rates, early miscarriage rates, and live birth rates (P>0.05). As shown in **Table 2**.

**Table 2:** Comparison of pregnancy outcomes in patients among three groups.

Item	HRT	NC	Down-regulation HRT	χ^2^ value	P value	Cramer’s Value	*Post Hoc* Power
Clinical pregnancy rate(%)	30.88(88/285)	47.50(95/200)^a^	40.38(42/104)	14.01	<0.001	0.154	0.927
Biochemical pregnancy rate(%)	35.09(100/285)	50.50(101/200)	45.19(57/104)	11.946	0.003	0.142	0.878
Early miscarriage rate(%)	21.59(19/88)	13.68(13/95)	21.43(9/42)	2.273	0.321	0.101	0.585
Live birth rate(%)	22.81(65/285)	36.50(73/200)^a^	30.77(32/104)	10.96	0.004	0.132	0.826

χ²: Chi−square test for categorical variables. Cramer’s V: Effect size for group differences.

^a^ represents P<0.05, the other two groups compared with the HRT group.

### 3.3 Logistic regression analysis based on pregnancy outcomes in patients among three groups

Multivariate logistic regression analysis was performed to adjust for factors including female body mass index, AMH, AFC, endometrial thickness on the day of transfer, the number of embryos transferred, and the type of embryos transferred.

As shown in **Table 3**, compared to the HRT group, the NC group had an increased clinical pregnancy rate (aOR=1.606, 95% CI: 1.063~2.427, P = 0.025) and biochemical pregnancy rate (aOR=1.532, 95% CI: 1.020-2.301, P = 0.040). There were no statistically significant differences in early miscarriage rate, and live birth rate (P>0.05) in these two groups. Additionally, there were no statistically significant differences in clinical pregnancy rate, biochemical pregnancy rate, early miscarriage rate, and live birth rate between HRT group and down-regulation HRT group (P>0.05).

**Table 3:** Logistic regression analysis of pregnancy outcomes in three groups.

Values	Clinical pregnancy rate		Biochemical pregnancy rate		Early miscarriage rate		Live birth rate	
	aOR(95%CI)	P value	aOR(95%CI)	P value	aOR(95%CI)	P value	aOR(95%CI)	P value
HRT group	Reference		Reference		Reference		Reference	
NC group	1.606(1.063~2.427)	0.025	1.532(1.020~2.301)	0.040	0.691(0.306~1.563)	0.375	1.422(0.909~2.224)	0.123
Down-regulation HRT group	1.270(0.774~2.083)	0.344	1.330(0.818~2.161)	0.250	1.240(0.480~3.199)	0.657	1.181(0.691~2.019)	0.542
Female BMI	1.006(0.949~1.067)	0.838	1.015(0.958~1.076)	0.611	1.006(0.892~1.135)	0.921	1.000(0.940~1.065)	0.988
AMH	1.004(0.998~1.009)	0.226	1.003(0.997~1.008)	0.347	1.003(0.995~1.012)	0.423	1.002(0.997~1.008)	0.393
AFC	1.020(0.987~1.054)	0.230	1.018(0.986~1.051)	0.263	0.952(0.893~1.014)	0.125	1.033(0.998~1.070)	0.069
Endometrial thickness on transfer day	1.333(1.173~1.514)	<0.001	1.302(1.148~1.476)	<0.001	0.704(0.530~0.935)	0.015	1.458(1.271~1.673)	<0.001
No. of embryo transferred	1.085(0.689~1.708)	0.726	1.020(0.654~1.593)	0.929	0.698(0.274~1.776)	0.451	1.147(0.702~1.875)	0.584
Type of embryo transferred	2.117(1.267~3.537)	0.004	2.184(1.325~3.601)	0.002	0.928(0.300~2.877)	0.897	1.849(1.056~3.236)	0.031

aOR, adjusted odds ratio in multivariate logistic regression. CI, confidence interval.

In covariate-adjusted analyses, female BMI, AMH, AFC, and the number of embryos transferred did not exert statistically significant effects on any of the pregnancy outcome measures (P > 0.05). The endometrial thickness on the day of embryo transfer emerged as an independent predictor for clinical pregnancy, biochemical pregnancy, and live birth, and early miscarriage (P<0.05). In addition, embryo type also exerted a significant positive impact on pregnancy outcomes, significantly improving the clinical pregnancy rate, biochemical pregnancy rate, and live birth rate (P<0.05).

## 4 Discussion

In assisted reproductive technology, the condition of the endometrium is one of the key factors determining the success of pregnancy assistance. Any factor affecting the integrity of the basal layer of the endometrium can lead to embryo implantation failure or early pregnancy loss. Intrauterine adhesion (IUA) is a common disease which would cause endometrial damage, with studies reporting that the incidence of secondary infertility in women with untreated IUA can be as high as 41% (1). The use of frozen-thawed embryo transfer (FET) in assisted reproductive technology is increasing, which requires endometrium preparation to establish receptivity to embryos. VNA et al. performed an open-label randomised controlled trial, which demonstrated that among patients with non-uterine factors undergoing FET, natural cycles, hormone replacement artificial cycles, and modified natural protocols yielded comparable pregnancy outcomes (2). More recently, a multicenter randomised study has further reported that natural cycles are associated with a higher live birth rate and a reduced early pregnancy loss rate, and patients with uterine cavity factors were also excluded in this investigation (3). However, the impact of different endometrial preparation protocols on FET-assisted pregnancy outcomes in patients after intrauterine adhesion separation surgery remains unclear. In the present study, a retrospective cohort analysis included 589 FET cycles was conducted, and multivariate logistic regression analysis revealed that among patients aged ≤35 years with a history of IUA surgery, the natural cycle (NC) protocol resulted in higher clinical pregnancy rate and biochemical pregnancy rate compared with the conventional hormone replacement therapy (HRT) protocol, with no significant differences observed between the NC protocol and the down-regulation-HRT protocol.

Mo et al. (4) used propensity score matching to compare the effects of HRT cycles and down-regulation HRT cycles on pregnancy outcomes in patients with intrauterine adhesions (IUA). The results indicated no difference in clinical pregnancy rates and early miscarriage rates between the two protocols, but the live birth rate was higher in the down-regulation HRT group compared to the HRT group. Wang et al. (5) conducted a real-world study suggesting that in the first FET cycle after hysteroscopic adhesiolysis, the natural cycle endometrial preparation protocol achieved a higher live birth rate compared to the artificial cycle and down-regulation artificial cycle protocols. A prospective cohort study by Yan et al. (6) indicated that patients with a history of intrauterine adhesions undergoing FET cycles with natural cycle endometrial preparation had similar clinical pregnancy rates to those without such a history, but the live birth rate was lower than in patients without a history of intrauterine adhesions. The inconsistencies in these results may be related to differences in study design, population inclusion criteria, and statistical analysis methods. The results of this study show some similarity to those of Wang et al., where, before adjusting for confounding factors, the natural cycle protocol group had higher clinical pregnancy, biochemical pregnancy rate and live birth rate compared with the HRT protocol; after adjustment, only the clinical pregnancy rate and biochemical pregnancy rate remained higher, while it appears to exhibit a trend toward improvement in the live birth rate without reaching statistical significance. Recent studies have shown that compared with women with no history of IUAs, women with a history of hysteroscopic adhesiolysis-treated IUAs were at higher risk of adverse obstetrical outcomes, such as pre-eclampsia, placenta accreta spectrum, and placenta previa (7, 8). These adverse outcomes, which often occur in the middle and late stages of pregnancy, are important factors leading to pregnancy loss and thus affect the final live birth rate. Although the adjustment of endometrial preparation protocols can optimize endometrial receptivity and improve the success rate of embryo implantation, it may not effectively intervene in the occurrence of subsequent adverse obstetrical outcomes related to IUA history. Furthermore, *post hoc* power analyze showed that the statistical power for detecting differences in live birth rate between groups might not be fully sufficient. This might be another potential reason why only a trend of improvement in live birth rate was observed without reaching statistical significance, rather than a true lack of effect of the endometrial preparation protocol on live birth rate.

It has been known that endometrial thickness is one of the key factors influencing embryonic implantation. In our study, the endometrial thickness on the transfer day was significantly higher in NC group than in HRT group. Wei et al. have also demonstrated that the patients in NC group had a higher endometrial thickness before FET (3). Logistic regression analysis further confirmed that endometrial thickness on the transfer day serves as an independent predictor of reproductive outcomes: each 1 mm increase in endometrial thickness on transfer day independently confers substantial benefits for reproductive outcomes, boosting clinical pregnancy, biochemical pregnancy and live birth rates by 33.3%, 30.2% and 45.8%, respectively, while reducing early miscarriage risk by 29.6%. These results suggest that NC protocol may offer benefits for promoting embryonic implantation.

The advantage of the natural cycle endometrial preparation protocol lies in the fact that the endometrium is naturally prepared for implantation during physiological processes, minimizing the intervention of exogenous medication. Recent studies have indicated that artificial cycles may not improve pregnancy outcomes in FET cycles and may instead increase the risk of adverse perinatal outcomes. Consequently, some scholars believe that regardless of the presence of ovulation disorders, artificial cycles should not be recommended as the first-choice protocol.

The number and type of embryos transferred represent critical determinants of pregnancy outcomes following FET (9). In the present study, we observed that several baseline characteristics, including the number and type of embryos transferred, exhibited significant imbalance across the study groups. These disparities are inherent to the retrospective study design, which may introduce confounding bias. After multivariable adjustment for these confounding factors, the number of embryos transferred did not demonstrate a significant independent association with any of the four outcome items. In contrast, the type of embryos transferred exerted a positive influence on pregnancy outcomes. Specifically, blastocyst transfer was associated with a significant increase in the clinical pregnancy rate, biochemical pregnancy rate, and live birth rate. These results suggest that compared with the number of embryos transferred, the type of embryos transferred may confer more clinical benefits for patients ≤35 years with a history of intrauterine adhesion separation surgery.

The strengths of the present study include its stringent inclusion and exclusion criteria, and the use of multivariate logistic regression analysis to adjust for confounding factors, which allowed for a comparison of the effects of various protocols on pregnancy outcomes, thereby enhancing the reliability of the conclusions. However, several limitations must be acknowledged. First, as a single-center retrospective study, the patient population was relatively homogenous, and treatment regimens may have been subject to institutional bias. Second, while *post hoc* power analysis revealed that the study was sufficiently powered to support the findings regarding clinical pregnancy, biochemical pregnancy, and live birth, a larger multi-center sample would be beneficial to further validate these results. Finally, the severity of IUA is a key factor affecting endometrial receptivity and clinical outcomes, and subgroup analysis stratified by IUA severity would provide more detailed evidence for clinical practice. However, subgroup analysis was not performed in the present study mainly due to the limited sample size.

In summary, for patients aged ≤35 years with a history of intrauterine adhesion surgery, using a natural cycle endometrial preparation protocol in frozen-thawed embryo transfer cycles can enhance clinical benefits for this cohort.

## Data Availability

The original contributions presented in the study are included in the article/Supplementary Material. Further inquiries can be directed to the corresponding author.

## References

[1] Schenker JG Margalioth EJ . Intrauterine adhesions: an updated appraisal. Fertil Steril. (1982) 37:593–610. doi: 10.1016/s0015-0282(16)46268-0. PMID: 6281085

[2] Vu NA Ho T Pham TD Nguyen NT Wang R Norman RJ . Livebirth rate after one frozen embryo transfer in ovulatory women starting with natural, modified natural, or artificial endometrial preparation in Viet Nam: an open-label randomised controlled trial. Lancet. (2024) 404:266–75. doi: 10.1016/s0140-6736(24)00756-6. PMID: 38944045

[3] Wei D Qin Y Sun Y Yan J Zhao H Guan Y . Natural ovulation versus programmed regimens before frozen embryo transfer in ovulatory women: multicentre, randomised clinical trial. BMJ. (2026) 392:e087045. doi: 10.1136/bmj-2025-087045. PMID: 41565309 PMC12821156

[4] Mo M Zheng Q Zhang H Xu S Xu F Wang Y . Hormone replacement therapy with GnRH agonist pretreatment improves pregnancy outcomes in patients with previous intrauterine adhesions. J Gynecol Obstet Hum Reprod. (2022) 51:102439. doi: 10.1016/j.jogoh.2022.102439. PMID: 35820621

[5] Wang C Peng Y Chen H Ban D Li Y Gong F . Analysis of clinical factors affecting live birth outcomes in the first FET cycle after intrauterine adhesion separation: a real-world study. Chin J Reprod Contraception. (2025) 45:45–58. doi: 10.3760/cma.j.cn101441-20240816-00300, PMID: 30704229

[6] Ouyang Y Peng Y Zheng M Mao Y Gong F Li Y . The impact of intrauterine adhesions on endometrial receptivity in patients undergoing *in vitro* fertilization-embryo transfer. Front Endocrinol (Lausanne). (2025) 15:1489839. doi: 10.3389/fendo.2024.1489839. PMID: 39906037 PMC11790458

[7] Hong W Wu Z Li L Wang B Li X . Intrauterine adhesions treated with hysteroscopic adhesiolysis and subsequent obstetric outcome: a retrospective matched cohort study. BJOG. (2025) 132:155–64. doi: 10.1111/1471-0528.17793. PMID: 38418403

[8] Mortimer RM Lanes A Srouji SS Waldman I Ginsburg E . Treatment of intrauterine adhesions and subsequent pregnancy outcomes in an *in vitro* fertilization population. Am J Obstet Gynecol. (2024) 231:536.e1–536.e10. doi: 10.1016/j.ajog.2024.05.026. PMID: 38777163

[9] Veleva Z Orava M Nuojua-Huttunen S Tapanainen JS Martikainen H . Factors affecting the outcome of frozen-thawed embryo transfer. Hum Reprod. (2013) 28:2425–31. doi: 10.1016/j.fertnstert.2009.07.206. PMID: 23756705

